# Ik2/TBK1 and Hook/Dynein, an adaptor complex for early endosome transport, are genetic modifiers of FTD-associated mutant CHMP2B toxicity in *Drosophila*

**DOI:** 10.1038/s41598-020-71097-5

**Published:** 2020-08-26

**Authors:** Yubing Lu, Ryan J. H. West, Marine Pons, Sean T. Sweeney, Fen-Biao Gao

**Affiliations:** 1 Department of Neurology, University of Massachusetts Medical School, Worcester, MA 01605 USA; 2 Sheffield Institute for Translational Neuroscience, University of Sheffield, Sheffield, S10 2HQ UK; 3 Neuroscience Institute, University of Sheffield, Sheffield, S10 2TN UK; 4 Department of Biology, University of York, York, YO10 5DD UK; 5 Present Address: Department of Pathology, Case Western Reserve University, Cleveland, OH 44106 USA

**Keywords:** Evolution, Neuroscience

## Abstract

Mutations in *CHMP2B*, encoding a protein in the endosomal sorting complexes required for transport (ESCRT) machinery, causes frontotemporal dementia linked to chromosome 3 (FTD3). FTD, the second most common form of pre-senile dementia, can also be caused by genetic mutations in other genes, including *TANK-binding kinase 1* (*TBK1*). How FTD-causing disease genes interact is largely unknown. We found that partial loss function of Ik2, the fly homologue of TBK1 also known as I-kappaB kinase ε (IKKε), enhanced the toxicity of mutant CHMP2B in the fly eye and that Ik2 overexpression suppressed the effect of mutant CHMP2B in neurons. Partial loss of function of Spn-F, a downstream phosphorylation target of Ik2, greatly enhanced the mutant CHMP2B phenotype. An interactome analysis to understand cellular processes regulated by Spn-F identified a network of interacting proteins including Spn-F, Ik2, dynein light chain, and Hook, an adaptor protein in early endosome transport. Partial loss of function of dynein light chain or Hook also enhanced mutant CHMP2B toxicity. These findings identify several evolutionarily conserved genes, including *ik2/TBK1*, *cut up* (encoding dynein light chain) and *hook,* as genetic modifiers of FTD3-associated mutant CHMP2B toxicity and implicate early endosome transport as a potential contributing pathway in FTD.

## Introduction

Frontotemporal dementia (FTD) is an early onset dementia associated with frontotemporal lobar degeneration (FTLD)^1^. Identified causative loci, collectively representing ~ 40% of all FTD cases, reveal a genetic, pathological and mechanistic overlap with amyotrophic lateral sclerosis (ALS)^2, 3^. Extensive cell biological studies of these loci suggest RNA metabolism (*TARDBP* and *FUS*) and autophagy/endosomal-lysosomal function (*CHMP2B*, *OPTN*, *p62*, *TBK1*, *Ubiquilin*-2, *VCP*) as major contributors to neuronal pathology^3, 4^. However, how different disease genes interact with each other in ALS/FTD pathogenesis remains poorly understood.

*CHMP2B* encodes a subunit of the endosomal sorting complex required for transport III (ESCRT-III) complex that is recruited to the surface of early endosomes to participate in the final step in membrane scission during the formation of multivesicular bodies (MVBs)^5^. MVBs are the endosomal and autophagosomal entry point to the late endosome and eventual lysosomal degradation. A splicing site mutation in *CHMP2B* resulting in a C-terminal truncation of the protein was identified in a Danish FTD patient cohort^6^ and other mis-sense mutations have since been identified in FTD and ALS pedigrees^7–12^. It is proposed that the truncation of the C-terminal in the CHMP2B^Intron5^ mutant protein promotes an ‘open’ configuration, locking the protein into an association with its binding partner Snf7-2/CHMP4B^13^. This blockage in ESCRT-III disassembly results in endosomal accumulation and deficient cellular trafficking^13–15^.

Why neurons are particularly susceptible to mutant CHMP2B induced endosomal dysfunction is not currently understood. To address this question, we established a *Drosophila* model expressing CHMP2B^Intron5^ post-mitotically in the fly eye^16^. Genetic screening for enhancers and suppressors of neurodegeneration in this model have so far identified activated innate immune signaling^16^, autophagosomal dysfunction^17^, and endosomal signaling disruption^18^ leading to JNK/AP-1 mediated pro-apoptotic signaling^18, 19^. Here, using our fly eye model of CHMP2B^Intron5^ mediated neurodegeneration, we attempt to identify additional genetic modifiers of mutant CHMP2B toxicity and effectors of endosomal dysfunction. In particular, we focus on some other genes known to be involved in ALS/FTD pathogenesis.

## Results

### Genetic interaction analysis identifies *ik2* as a strong genetic modifier of mutant CHMP2B toxicity in *Drosophila*

**Figure 1:**
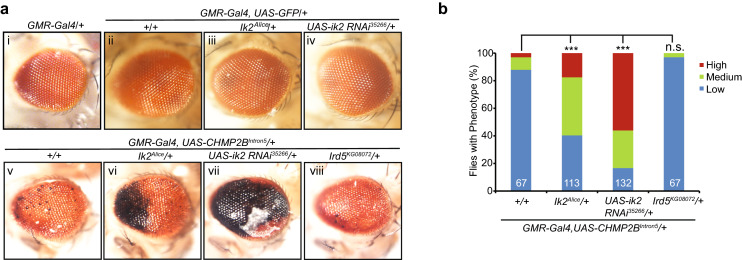
Genetic interactions between *CHMP2B*^*Intron5*^ and *ik2*, the fly homologue of mammalian *TBK1*, a gene mutated in a subset of ALS/FTD patients. (**a**) Compared to control flies (i, ii), the majority of 1-day-old *GMR-Gal4,UAS-CHMP2B*^*Intron5*^*/CyO* flies had a weak eye degeneration phenotype (v). Partial loss of Ik2 activity through the *ik2*^*Alice*^ allele (iii) or RNAi knockdown (iv) did not cause an eye phenotype but significantly enhanced the *CHMP2B*^*Intron5*^ eye phenotype (vi, vii). In contrast, partial loss of function in *ird5*, another fly homologue of mammalian *TBK1*, had no additional effect (viii). The eye images were taken with a Nikon DS-Fi1 camera on a Nikon SMZ1500 stereomicroscope using the NIS-Element BR software version 3.10. This software requires purchase and is not freely available. (**b**) Quantification of eye phenotypes in flies of different genotypes in panel a by categorical data analysis. ***p < 0.001 by Chi-square test for three categorical variables (Low, Medium and High). n.s.: not significant.

We previously generated a fly model of FTD-associated mutant CHMP2B neurotoxicity, in which expression of CHMP2B^Intron5^ but not CHMP2B^WT^ driven by *GMR-Gal4* produced a retinal degeneration phenotype characterized by a few small melanin deposits in the fly eye^16^. Here we first confirmed this eye phenotype in flies expressing CHMP2B^Intron5^ (Fig. 1a/v) but no phenotype when expressing GFP (Fig. 1a/i). To identify what other ALS/FTD genes may genetically interact with mutant CHMP2B, we tested mutants of *ter94* and *ik2*, fly homologues of VCP and TBK1, respectively, for their ability to dominantly modify the phenotype of fly eyes expressing CHMP2B^Intron5^. We found partial loss of either ter94 (not shown) or *ik2* gene were enhancers of mutant CHMP2B toxicity (Fig. 1a). The latter is the focus of the current study. The *ik2* gene is also known as I-kappaB kinase ε (IKKε) in Flybase. A point mutation named *Alice* (*ik2*^*Alice*^) compromises the normal function of *ik2*^20^. *Ik2*^*Alice*^ heterozygous flies did not show any eye degeneration phenotype (Fig. 1a-iii). However, mutant CHMP2B toxicity was significantly enhanced in the *ik2*^*Alice*^ heterozygous background (Fig. 1a/vi,b), suggesting that *ik2* is a strong genetic modifier of mutant CHMP2B toxicity in the fly eye. This finding was further confirmed by *ik2* RNAi knockdown. The *UAS-ik2 RNAi*^*35266*^ line was used and validated by others before^21^. Expression of *ik2* RNAi by itself did not cause an eye phenotype (Fig. 1a/iv) but enhanced the toxicity of mutant CHMP2B (Fig. 1a/vii) to a much greater extent than the *ik2*^*Alice*^ allele (Fig. 1b), presumably reflecting a greater knockdown of Ik2 activity. To demonstrate the specificity of the genetic interaction between *ik2* and mutant CHMP2B, we also examined the effect of partial loss of IRD5 activity, another member of the *Drosophila* I kappa B kinase (IKK) family. The *Ird5*^*KG08072*^ allele did not modify the eye phenotype of mutant CHMP2B (Fig. 1a/viii,b). Thus, partial loss of Ik2 function in the presence of mutant CHMP2B may compromise the same cellular pathway leading to neurodegeneration.

**Figure 2:**
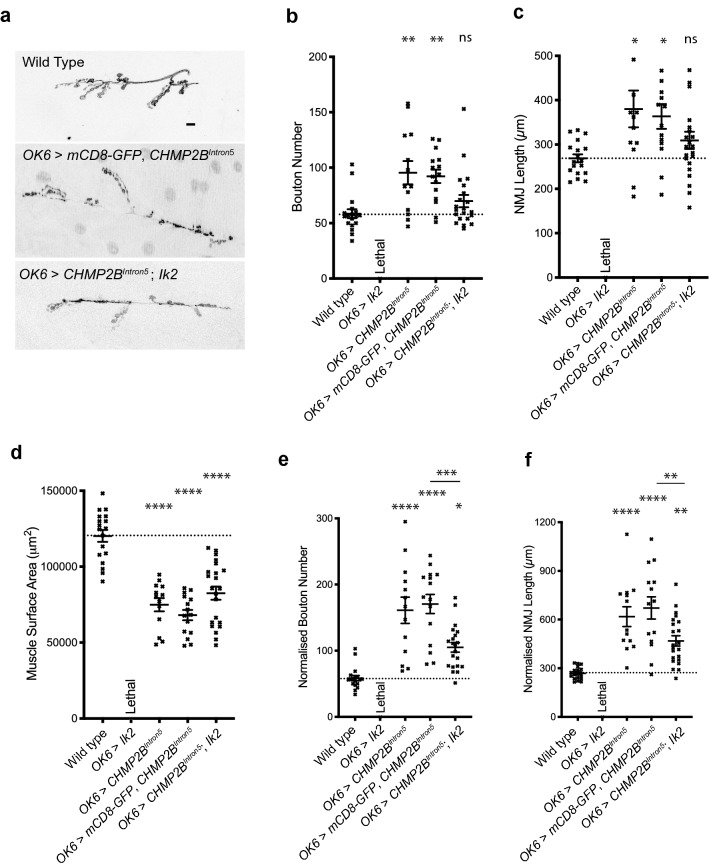
Co-expression of Ik2 alleviates CHMP2B^Intron5^-dependent overgrowth at the *Drosophila* larval neuromuscular junction (NMJ). (**a**) Representative micrographs of NMJs at muscle 6/7, hemi-segment A3 in 3rd instar larvae. Scale bar: 10 μm. Leica MM AF Premier Version 1.5.0 software (https://www.leica-microsystems.com/products/microscope-software/) was used for imaging. Graphs were made in Graphpad prism 8 (https://www.graphpad.com/scientific-software/prism/). Both softwares are not free to use. (**b**,**c**) Increase in synaptic bouton number (**b**) and NMJ length (**c**) associated with expression of *UAS-CHMP2B*^*Intron5*^ in motor neurons can be ameliorated through co-expression of *UAS-ik2*, but not *UAS-mCD8-GFP*. Muscle 6/7, hemi-segment A3, 3rd instar larvae. ANOVA with Tukey’s post-hoc multiple comparison test**p < 0.01 by ANOVA with Tukey’s post-hoc multiple comparison test. (**d**) Co-expression of *UAS-ik2* has no effect on the reduced muscle sizes in larvae expressing *UAS-CHMP2B*^*Intron5*^ in motor neurons. Muscle 6/7, hemi-segment A3, 3rd instar larvae. ****p < 0.0001 by ANOVA with Tukey’s post-hoc multiple comparison test. (**e**,**f**) Normalization of bouton number (**e**) and NMJ length (**f**) to account for significantly reduced muscle sizes. Muscle 6/7, hemi-segment A3, 3rd instar larvae. *p < 0.05, **p < 0.01, ***p < 0.001 and ****p < 0.0001 by ANOVA with Tukey’s post-hoc multiple comparison test.

Ik2 shares a higher identity with TBK1 (35.5%) than with other human kinases such as IKK-epsilon and IKK-beta (34.5% and 18.7%, respectively). Human TBK1 shares 35.5% and 18.5% identity with Ik2 and IRD5, respectively. Thus, Ik2 appears to be the *Drosophila* homologue of human TBK1. Partial loss of TBK1 activity causes both ALS and FTD^22, 23^. To further examine the genetic interaction between *Ik2/TBK1* and mutant CHMP2B, we also used the *Drosophila* neuromuscular junction (NMJ) as the experimental system. Previously we reported that pan-neuronal expression of mutant CHMP2B resulted in a significant overgrowth at the *Drosophila* third instar larval NMJ^18, 19^. Here we demonstrate that this synaptic overgrowth phenotype was also observed when CHMP2B^Intron5^ is expressed specifically in *Drosophila* motor-neurons, under the control of the *OK6-Gal4* motorneuron driver. Motor-neuronal expression of CHMP2B^Intron5^ resulted in a significant increase in bouton number and NMJ length coupled with a significant decrease in muscle surface area (Fig. 2a–d) and decline in overall motor function (Supplementary 2). While motor-neuronal expression of *UAS-ik2* resulted in lethality, co-expression of Ik2 with CHMP2B^Intron5^ was not lethal, presumably because the dilution of Gal4 by two UAS elements leading to a lower level of ectopic Ik2 expression. IK2 expression was sufficient to rescue CHMP2B^Intron5^ dependent synaptic overgrowth, but co-expression of mCD8-GFP with CHMP2B^Intron5^ had no effect. Normalization of NMJ lengths and bouton numbers against muscle surface area reveals that Ik2 rescue of the CHMP2B^Intron5^ phenotypes appears to be independent of altered muscle surface area (Fig. 2e,f).

### *Spn-F* is a strong genetic modifier of mutant CHMP2B toxicity

**Figure 3:**
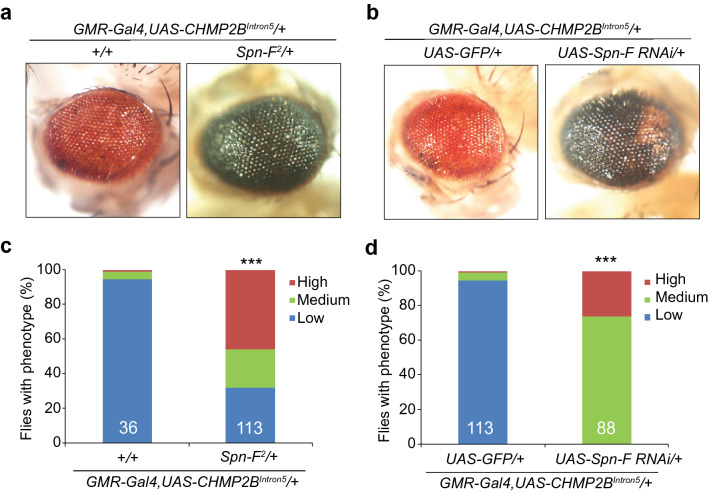
Partial loss of function of *Spn-F*, a phosphorylation target of Ik2, enhances the toxicity of *CHMP2B*^*Intron5*^ in vivo. (**a**) Partial loss of Spn-F activity greatly enhanced the *CHMP2B*^*Intron5*^ eye phenotype, as judged by comparison with control flies. (**b**) RNAi mediated knockdown of Spn-F activity also enhanced the *CHMP2B*^*Intron5*^ eye phenotype. The eye images were taken with a Nikon DS-Fi1 camera on a Nikon SMZ1500 stereomicroscope using the NIS-Element BR software version 3.10. This software requires purchase and is not freely available. (**c**,**d**) Quantification of the eye phenotypes in panel **a** (**c**) and panel **b** (**d**) by categorical data analysis. ***p < 0.001 by Chi-square test for three categorical variables (Low, Medium and High). n.s.: not significant.

Spindle F (Spn-F) is a major phosphorylation target of Ik2, and these two proteins form a complex that regulates several developmental processes in *Drosophila*^24–26^*.* Therefore, we examined whether Spn-F is also a genetic modifier of mutant CHM2B toxicity. Indeed, the toxicity of mutant CHMP2B in the eye was greatly enhanced in the *Spn-F*^*2*^ heterozygous background (Fig. 3a,c); this finding was further confirmed by RNAi-mediated reduction of Spn-F activity (Fig. 3b,d), while flies with *Spn-F* RNAi knockdown did not show eye degeneration phenotypes (Supplementary 3).

### Hook interacts with Spn-F

To determine which cellular pathways regulated by the Ik2-Spn-F complex are involved in mutant CHMP2B toxicity, we sought to identify other interacting proteins by immunoprecipitation of Spn-F followed by mass spectrometry. We expressed *UAS-EGFP-Spn-F* using *actin-Gal4* and used GFP antibody to pull down Spn-F from fly head lysates. We identified several proteins that bind to Spn-F (Table 1). The most abundant was Ik2, validating the experimental approach, which also identified Hook and dynein light chain 1 (Dlc1), which is encoded by the gene *cut up* (*ctp*). Although the functional homolog of Spn-F in mammals is unknown, both Hook and Dlc1 are highly conserved evolutionarily^27, 28^. Spn-F directly interacts with Dlc1 in flies^29^ and Hook family proteins activate dynein adaptors in mammalian early endosome transport^30^. Thus, our interactome analysis identified Hook as another protein that interacts with Spn-F.

**Table 1:** Spn-F interacting proteins identified by mass spectrometry.

Protein name	Flybase symbol	Accession number	Molecular weight (kDa)	Total reads	
				GFP-SpnF	GFP
Spindle-F	spn-F	A0A0B4KI68	42	52	0
I-kappaB kinase ε	IKKε	Q7KJQ4	81	17	0
Hook	Hook	Q24185	77	5	0
Cut up	cpt	Q24117	10	4	0
Yolk protein 1	Yp1	P02843	49	3	0
Serine-arginine protein 55	B52	P26686	43	2	0
Histone H4	His4	P84040	11	3	0
Triosephosphate isomerase	Tpi	P29613	27	2	0
Cytochrome c oxidase subunit 4	CoI4	Q9VIQ8	21	2	0

### *Hook* and *ctp* are also strong genetic modifiers of mutant CHMP2B toxicity

**Figure 4:**
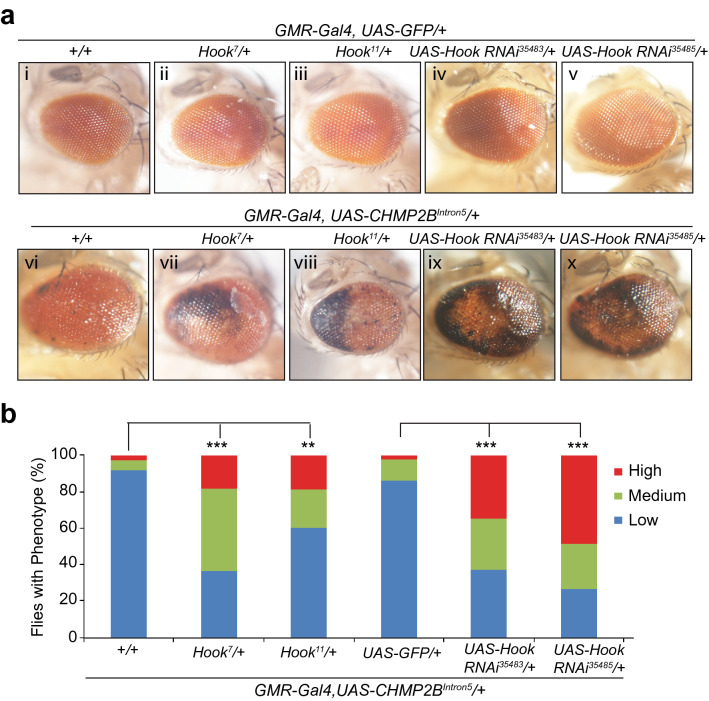
Partial loss of function of Hook enhances the neurotoxicity of *CHMP2B*^*Intron5*^ in vivo. (**a**) As judged by comparison with control flies (i), multiple *hook* genetic alleles (ii, iii) or RNAi knockdown (iv, v) in the eye did not cause any eye phenotypes. In contrast, comparison with flies expressing *CHMP2B*^*Intron5*^ in the eye (vi) showed that partial loss of function of *hook* through genetic alleles (vii, viii) or different RNAi lines (ix, x) greatly enhanced the toxicity of *CHMP2B*^*Intron5*^. The eye images were taken with a Nikon DS-Fi1 camera on a Nikon SMZ1500 stereomicroscope using the NIS-Element BR software version 3.10. This software requires purchase and is not freely available. (**b**) Quantification of eye phenotypes in panel a by categorical data analysis. **p < 0.01, ***p < 0.001, by chi-squared test for three categorical variables (Low, Medium and High).

**Figure 5:**
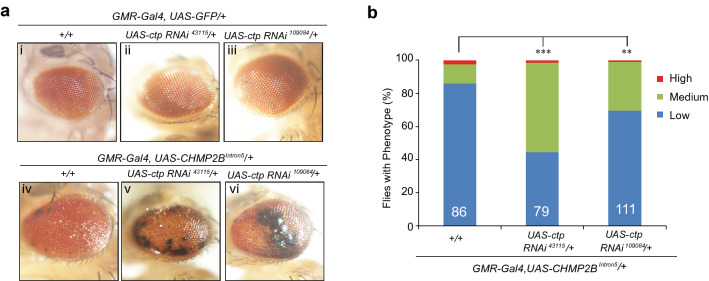
Partial loss of function of *ctp* also enhances the neurotoxicity of mutant CHMP2B in vivo. (**a**) Eye morphology in flies expressing two different *ctp*-specific RNAi lines (ii, iii) was indistinguishable from that of control flies (i). Comparison with flies expressing mutant CHMP2B (iv) showed that partial loss of function of *ctp* through RNAi-mediated knockdown (v, vi) enhanced mutant CHMP2B toxicity. The eye images were taken with a Nikon DS-Fi1 camera on a Nikon SMZ1500 stereomicroscope using the NIS-Element BR software version 3.10. This software requires purchase and is not freely available. (**b**) Quantification of eye phenotypes in (**a**) by categorical data analysis. ***p < 0.001, **p < 0.01, by Chi-square test for three categorical variables (Low, Medium and High).

Because Hook has a specific role in early endosome transport^30^, we sought to determine whether this cellular pathway contributes to the toxicity of mutant CHMP2B. For this analysis, we used two *hook* mutant alleles (*hook*^*7*^ and *hook*^*11*^) and two *hook*-specific RNAi lines (Fig. 4a). As judged by comparison with control flies (Fig. 4a/i), *Hook* heterozygous mutant flies (Fig. 4a/ii, iii) or flies expressing *hook* RNAi (Fig. 4a/iv,v) appeared to have normal morphology. However, partial loss of Hook activity, through genetic alleles (Fig. 4a/vii,viii) or RNAi knockdown (Fig. 4a/ix,x), greatly enhanced the retinal degeneration phenotype caused by mutant CHMP2B (Fig. 4a/vi,b). We also used two different RNAi lines to knockdown *ctp* expression, at least one of these RNAi lines has been previously characterized by others^31^. We found that RNAi knockdown of *ctp* activity also worsened this phenotype (Fig. 5). Thus, compromised early endosome transport contributes to neurodegeneration induced by FTD3-associated mutant CHMP2B.

## Discussion

**Figure 6:**
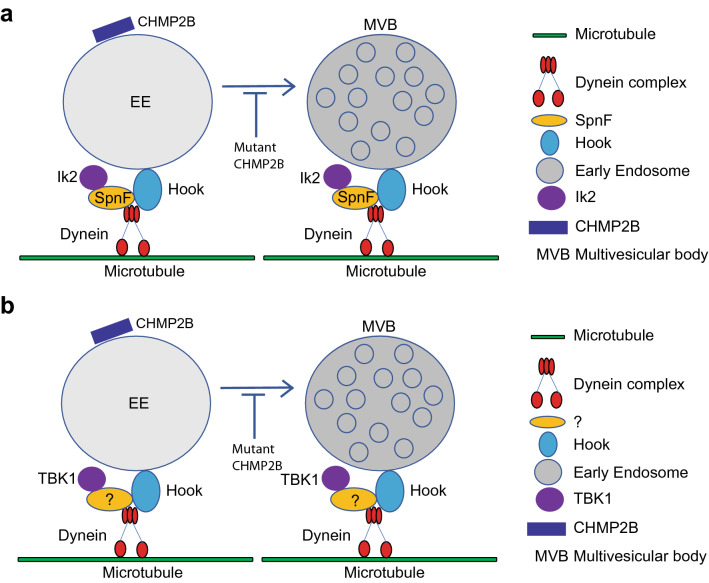
Schematic representation of endosome transport on microtubules mediated by the Hook-Dynein complex in flies (**a**) and mammalian cells (**b**). The mammalian equivalent of Spn-F remains to be identified. Moreover, how endogenous Ik2/TBK1 and Hook family proteins interact in flies and mammalian neurons needs to be further investigated.

Through genetic interaction analysis, we first identified the genes encoding Ik2 and its known binding target, Spn-F, as strong genetic modifiers of mutant CHMP2B toxicity in *Drosophila*. We then identified Hook as another protein that interacts with Spn-F by immunoprecipitation and mass spectrometry analyses. Exactly how endogenous Ik2/TBK1 and Hook family proteins interact in flies and mammalian neurons needs to be further investigated (Fig. 6). Further genetic studies indicated that the genes encoding Hook, an adaptor molecule for early endosome transport, and its binding partner Dlc1 are also strong modifiers of mutant CHMP2B toxicity. Together, our studies identified three evolutionarily conserved genes, *ik2*, *hook* and *ctp*, as previously unknown genetic modifiers of FTD3-associated mutant CHMP2B and suggest that compromised early endosome transport contributes to neurodegeneration in FTD (Fig. 6).

CHMP2B is a subunit of ESCRT-III required for the maturation of early endosomes into MVBs^5^. As expected, this process is disrupted by the ectopic expression of FTD3-associated mutant CHMP2B, leading to a disruption of intracellular sorting or degradation of cargo proteins such as EGF receptor^13^ and accumulation of aberrant Rab7-positive endosomal structures^13, 14^. Mutant CHMP2B acts through its failure to dissociate from ESCRT-III due to its lack of C-terminus that is required to interact with SKD1, an AAA family ATPase essential for ESCRT-III dissociation^13, 32^. Indeed, expression of a dominant-negative form of SKD1 caused a similar endosomal phenotype as that induced by mutant CHMP2B^13^. Moreover, mutant CHMP2B disrupts regulation of TGF-β and JNK signaling in the endosome^18^. Thus, abnormal endosomal function is a key pathological mechanism in FTD3. This notion is further supported by our finding here that partial loss of function in the Hook-Dlc1 complex required for early endosome transport greatly exacerbates mutant CHMP2B toxicity. Taken together, these studies suggest that normalizing endosomal function is a promising potential therapeutic approach for FTD3.

## Conclusion

Genetic analyses in a *Drosophila* model of FTD3 identify several evolutionarily conserved genes, including *ik2/TBK1*, *ctp/Dlc1* and *hook,* as genetic modifiers of mutant CHMP2B toxicity. These findings implicate early endosome transport as a potential contributing pathway and, together with earlier reports, suggest that normalizing endosomal function is a promising potential therapeutic approach for FTD3.

## Materials and methods

### Fly strains and maintenance

Flies were raised at 25 °C on a standard diet. *GMR-Gal4, UAS-CHMP2B*^*Intron5*^ recombined flies were generated and studied previously^16^. *GMR-Gal4, UAS-GFP, ik2*^*Alice*^*, UAS-ik2 RNAi, ird5*^*KG08072*^*, UAS-Spn-F RNAi* and *Ter94*^*K15502*^/*CyO *were from the Bloomington *Drosophila* Stock Center. *GMR-Gal4, UAS-GFP* recombined flies were generated in this study. *UAS-hook RNAi* (35483 and 35485)*,* and *UAS-ctp RNAi* (43115 and 104084) were from the Vienna *Drosophila* RNAi Center. *Spn-F*^*2*^ and *UAS-GFP-Spn-F* fly lines^33^ were kindly provided by Dr. Hsiu-Hsiang Lee, and *hook*^*7*^ and *hook*^*11*^ fly lines^34^ were from Dr. Helmut Krämer. The genetic aberrations of these alleles are summarized in Supplementary 1. For genetic interaction studies, the recombined stock, *GMR-Gal4, UAS-CHMP2B*^*Intron5*^/*CyO*, was crossed with individual classic mutants or RNAi lines. To quantify the *CHMP2B*^*Intron5*^ eye phenotype, we arbitrarily classified the eye phenotype with or without enhancers into three groups based on the relative abundance of black spots on the surface of the eye.

### Immunoprecipitation, SDS-PAGE, and silver stain

Adult *Actin-Gal4/UAS-GFP* and *Actin-Gal4/UAS-GFP-Spn-F* flies were frozen with dry ice and vortexed to remove the heads. Heads from each genotype were homogenized in lysis buffer (50 mM Tris–HCl, pH 7.5, 150-mM sodium chloride, 1% Nonidet P40, 0.5% sodium deoxycholate, 1 tablet of complete Mini protein inhibitor cocktail/10 mL). Homogenates were centrifuged at 4 °C for 20 min at 12,000*g*. Protein concentrations were determined with the Bradford assay (Bio-Rad). For co-immunoprecipitation experiments, supernatants of GFP and GFP-Spn-F with the same amount of total proteins were incubated with GFP magnetic beads (Chromoteck) overnight at 4 °C. The beads were incubated, washed three times for 15 min each with washing buffer (10 mM Tris–HCl, pH 8.0, 150 mM NaCl, 0.1% Nonidet P40), and then suspended in the gel loading buffer and boiled for 5 min. The co-immunoprecipitation samples were then run on a 10% polyacrylamide-SDS gel for a short time, and stained with a silver staining kit (Sigma) for subsequent digestion and downstream LC–MS/MS analysis (Proteomics and Mass Spectrometry Facility at UMass).

### Neuromuscular junction (NMJ) analysis

For NMJ analysis, *Drosophila* were raised on standard cornmeal medium at 18 °C on a 12-h light:dark cycle. Immunohistochemistry was performed described^20^. Motor-neuronal expression was under the control of the *OK6-Gal4* driver. NMJs were imaged at 40× with a Hamamatsu ORCA-R2 C10600-10B digital camera on a Leica DM6000B microscope fitted with Qioptiq OptiGrid Structured-Light system using Leica MM AF software. Muscles were imaged with the same system at 10x, without the OptiGrid. NMJ analysis was done as described^18^. Prism 7 (GraphPad Software) was used for statistical analysis.

### LC–MS/MS protein identification

This analysis was performed by UMass Proteomics Core Facility and Dr. John Leszyk provided the method description that was previously published^35, 36^.

#### In gel digestion

Silver-stained gel bands were destained with a 1:1 ratio of potassium ferricyanide (30 mM) and sodium thiosulfate (100 mM). Gels were washed extensively with water to remove and destain the yellow color. Gel slices were cut into 1 × 1-mm pieces and placed in 1.5-ml eppendorf tubes with 1 ml of water for 30 min. The water was removed, and 200 µl of 250 mM ammonium bicarbonate was added. For reduction, 25 µl of a 45-mM solution of 1,4 dithiothreitol was added, and the samples were incubated at 50 °C for 30 min. After cooling to room temperature, the samples were alkylated by adding 25 µl of a 100-mM iodoacetamide solution for 30 min. The gel slices were washed twice with 1-ml aliquots of water. The water was removed, and 1 ml of a 50:50 mixture of 50-mM ammonium bicarbonate and acetonitrile was placed in each tube. After incubation at room temperature for 1 h, the solution was removed, and 200 µl of acetonitrile was added to each tube, turning the gel slices opaque white. The acetonitrile was removed, and the gel slices were further dried in a Speed Vac and rehydrated in 1,000 µl of 2 ng/µl trypsin (Sigma) in 0.01% ProteaseMAX Surfactant (Promega):50-mM ammonium bicarbonate. Samples were incubated at 37 °C for 21 h. The supernatant of each sample was removed and placed in a separate 1.5-ml eppendorf tube. Gel slices were further dehydrated with 100 µl of an 80:20 mixture of acetonitrile and 1% formic acid. The extract was combined with the supernatants of each sample. The samples were then dried in a Speed Vac.

#### LC/MS/MS on Q exactive

After reconstitution in 25 µl of 0.1% trifluoroacetic acid in 5% acetonitrile, a 3-µl aliquot of each sample was directly injected onto a custom-packed 2 cm × 100 µm C_18_ Magic 5-µm particle trap column. Peptides were eluted and sprayed from a custom-packed emitter (75 µm × 25 cm C_18_ Magic 3-µm particle) with a linear gradient from 95% solvent A (0.1% formic acid in water) to 35% solvent B (0.1% formic acid in acetonitrile) for 90 min at a flow rate of 300 nanoliters per minute on a Waters Nano Acquity UPLC system. Data-dependent acquisitions were done on a Q Exactive mass spectrometer (Thermo Scientific) according to an experiment in which full MS scans from 300 to 1,750 m/z were acquired at a resolution of 70,000 followed by 10 MS/MS scans acquired under higher-energy collisional dissociation (HCD) fragmentation at a resolution of 17,500 with an isolation width of 1.6 Da. Raw data files were processed with Proteome Discoverer (Thermo, version 1.4) and then searched with Mascot Server (Matrix Sciences, version 2.5) against the human index of Uniprot. The search parameters used were fully tryptic with 2 missed cleavages, parent mass tolerances of 10 ppm, and fragment mass tolerances of 0.05 Da. Variable modifications of acetyl (protein N-term), pyro glutamic for N-term glutamine, oxidation of methionine, and carboxymethyl cysteine were considered.

### Statistical analysis

Significant difference between control and experimental groups were determined by using Chi-square tests to calculate P values for categorical data.

### Other information

Detailed information is also provided regarding genotypes of flies (Supplementary 4) and reagents (Supplementary 5) as well as values of all the statistical rest results (Supplementary 6).

## Supplementary information

Supplementary Information

## Data Availability

The datasets generated during and/or analysed during the current study are available from the corresponding author on reasonable request.
