# Supplementary material for: Ik2/TBK1 and Hook/Dynein, an adaptor complex for early endosome transport, are genetic modifiers of FTD-associated mutant CHMP2B toxicity in Drosophila
Source: Sci Rep. 2020 Aug 26;10:14221. doi: 10.1038/s41598-020-71097-5 (PMC7450086; doi:10.1038/s41598-020-71097-5)
Supplement: Supplementary file 1 — Supplementary Information [file 41598_2020_71097_MOESM1_ESM.pdf]

Supplementary Materials

Ik2/TBK1 and Hook/Dynein, an adaptor complex for early endosome transport, are genetic modifiers of FTD-associated mutant CHMP2B toxicity in *Drosophila*

Yubing Lu[1,#], Ryan JH West[2,3], Marine Pons[1], Sean T. Sweeney[4], Fen-Biao Gao[1,*]

[1] Department of Neurology, University of Massachusetts Medical School, Worcester, MA 01605, USA

[2] Sheffield Institute for Translational Neuroscience, University of Sheffield, Sheffield, S10 2HQ, UK

[3] Neuroscience Institute, University of Sheffield, Sheffield, S10 2TN, UK

[4] Department of Biology, University of York, York, YO10 5DD, UK

Supplementary 1. Genetic aberrations of alleles used in this study.

Supplementary 2. Motor function defect in *CHMP2B*^{Intron5} Larvae

Motor neuronal expression of *UAS-CHMP2B*^{Intron5} by *OK6-Gal4* results in a significant decrease in median crawling speed in 3rd instar wandering larvae compared to wild-type (Canton S, w^{1118} outcross) and driver only (*OK6-Gal4*/+) larvae. Co-expression of *UAS-ik2* shows no significant difference to *UAS-CHMP2B*^{Intron5} larvae, indicating other pathways also contribute to motor dysfunction induced by mutant CHMP2B. ANOVA with Tukey's post-hoc multiple comparison to wild-type **: $p < 0.01$, ***: $p < 0.001$.

Supplementary 3. Partial loss of Spn-F activity does not induce retinal degeneration phenotype.

A genetic allele and UAS-RNAi were used to knockdown Spn-F in the eye. The image was taken with a Nikon DS-Fi1 camera on a Nikon SMZ1500 stereomicroscope using the NIS-Element BR software version 3.10. This software requires purchase and is not freely available. However, a simpler software, NIS-Elements Viewer, is freely available (https://www.microscope.healthcare.nikon.com/en_AOM/products/software/nis-elements/viewer).

Supplementary 4. Genotypes of the flied used in this study.

Supplementary 5. Fly lines and reagents used in this study and their sources.

Supplementary 6. Values of all the statistical test results.

Methods

Motor function assay

Larval locomotor assays were performed as described previously [1]. Briefly, female third instar wandering larvae, raised at 18 °C, of the appropriate genotype were selected and washed in HL3 (70 mM NaCl, 5 mM KCl, 1 mM $CaCl_2{\cdot}2H_2O$, 10 mM $NaHCO_3$, 5 mM trehalose, 115 mM sucrose and 5 mM BES in dH_2O). Larvae were filmed from above for 120 s (0.2 frames s^{-1}) using VirtualDub software whilst crawling on a petri dish filled with 1% agar, 2-3 at a time. Videos were analyzed using imageJ and custom macros using the MTrack2 plugin as described [1] .

References

1. Ahmad, S. T., Sweeney, S. T., Lee, J. A., Sweeney, N. T. & Gao, F. B. Genetic screen identifies serpin5 as a regulator of the toll pathway and CHMP2B toxicity associated with frontotemporal dementia. *Proceedings of the National Academy of Sciences of the United States of America* **106**, 12168-12173 (2009).
2. Bergalet, J. *et al.* Inter-dependent Centrosomal Co-localization of the cen and ik2 cis-Natural Antisense mRNAs in Drosophila. *Cell reports* **30**, 3339-3352 e3336, (2020).
3. Lin, T. *et al.* Spindle-F Is the Central Mediator of Ik2 Kinase-Dependent Dendrite Pruning in Drosophila Sensory Neurons. *PLoS genetics* **11**, e1005642 (2015).
4. Kramer, H. & Phistry, M. Genetic analysis of hook, a gene required for endocytic trafficking in drosophila. *Genetics* **151**, 675-684 (1999).

5. West RJH, Ugbode C, Gao FB, Sweeney ST. The pro-apoptotic JNK scaffold POSH/SH3RF1 mediates CHMP2BIntron5-associated toxicity in animal models of frontotemporal dementia. Hum Mol Genet 27: 1382-1395 (2018).
6. Zaytseva, O. *et al.* The novel zinc finger protein dASCIZ regulates mitosis in Drosophila via an essential role in dynein light-chain expression. *Genetics* **196**, 443-453 (2014).

Fly mutants	Mutation information
Ik2^{Alice}	Point mutation in the kinase domain (E297I)
Ird5^{KG08072}	P element intertion mutation
Ter94^{K15502}	P element intertion mutation
Spn-F^{2}	A 67 nucleotide deletion starting at base 726 (where +1 is the A of the initiator Met)
Hook^{7}	Point mutation, resulting in a 185aa polypeptide.
Hook^{11}	Point mutation, resulting in a 60aa polypeptide.

Supplementary 1. Genetic aberrations of alleles used in this study.

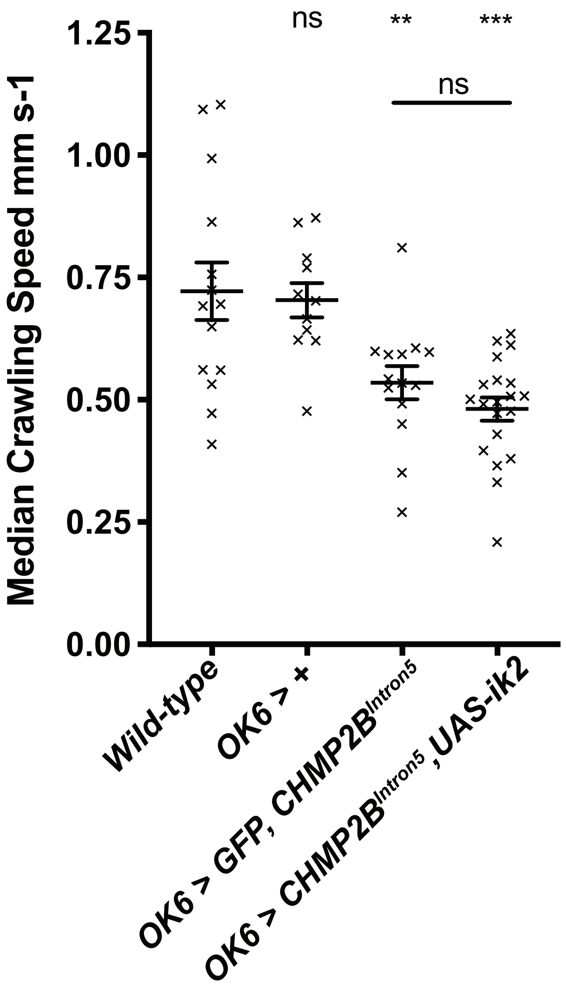

Supplementary 2. Motor function defect in CHMP2BIntron5 Larvae
Motor neuronal expression of *UAS-CHMP2BIntron5* by *OK6-Gal4* results in a significant decrease in median crawling speed in 3rd instar wandering larvae compared to wild-type (Canton S, *w1118* outcross) and driver only (*OK6-Gal4*/+) larvae. Co-expression of *UAS-ik2* shows no significant difference to UAS-CHMP2BIntron5 larvae, indicating other pathways also contribute to motor dysfunction induced by mutant CHMP2B. ANOVA with Tukey's post-hoc multiple comparison to wild-type **: p <0.01, ***: p <0.001.

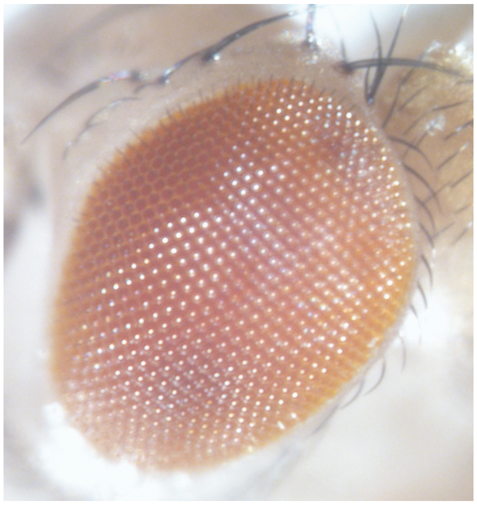

GMR-Ga4;UAS-Spn-F RNAi

Supplementary 3. Partial loss of Spn-F activity does not induce retinal degeneration phenotype. A UAS-RNAi was used to knockdown Spn-F in the eye.

Associated figures	Genotypes
**Figure 1**	*+/w^{1118}; GMR-Gal4/+;+/+*
	+/w^{1118}; GMR-Gal4, UAS-GFP/+;+/+
	+/w^{1118}; GMR-Gal4, UAS-GFP/Ik2^{Alice};+/+
	+/w^{1118}; GMR-Gal4, UAS-GFP/+;UAS-Ik235266/+
	+/w^{1118}; GMR-Gal4, UAS-CHMP2B^{Intron5}/+;+/+
	+/w^{1118}; GMR-Gal4, UAS-CHMP2B^{Intron5}/Ik2^{Alice};+/+
	+/w^{1118}; GMR-Gal4, UAS-CHMP2B^{Intron5}/+; UAS-Ik2 RNAi^{235266}/+
	+/w^{1118}; GMR-Gal4, UAS-CHMP2B^{Intron5}/+; Ird5^{KG08072}/+
**Figure 2**	+/*w^{1118}*;+/+;+/+ (Outcrossed Wild-type)
	+/w^{1118};OK6-Gal4/+;+/+
	+/w^{1118};OK6-Gal4/UAS-CHMP2B^{Intron5};+/+
	+/w^{1118};OK6-Gal4/+;UAS-ik2/+
	+/w^{1118};OK6-Gal4/UAS-CHMP2B^{Intron5};UAS-ik2/+
**Figure 3**	*+/w^{1118}; GMR-Gal4, UAS-CHMP2B^{Intron5}/+;+/+*
	+/w^{1118}; GMR-Gal4, UAS-CHMP2B^{Intron5}/+;Spn-F^{2}/+
	+/w^{1118}; GMR-Gal4, UAS-CHMP2B^{Intron5}/UAS-GFP;+/+
	+/w^{1118}; GMR-Gal4, UAS-CHMP2B^{Intron5}/UAS-Spn-F RNAi^{65226};+/+
**Figure 4**	*+/w^{1118}; GMR-Gal4, UAS-GFP/+;+/+*
	+/w^{1118}; GMR-Gal4, UAS-GFP/Hook^{7};+/+
	+/w^{1118}; GMR-Gal4, UAS-GFP/Hook^{11};+/+
	+/w^{1118}; GMR-Gal4, UAS-GFP/+^{1};UAS-Hook RNAi^{35483}/+
	+/w^{1118}; GMR-Gal4, UAS-GFP/+^{1};UAS-Hook RNAi^{35485}/+
	+/w^{1118}; GMR-Gal4, UAS-CHMP2B^{Intron5}/+;+/+
	+/w^{1118}; GMR-Gal4, UAS-CHMP2B^{Intron5}/Hook^{7};+/+
	+/w^{1118}; GMR-Gal4, UAS-CHMP2B^{Intron5}/Hook^{11};+/+
	+/w^{1118}; GMR-Gal4, UAS-CHMP2B^{Intron5}/+^{1};UAS-Hook RNAi^{35483}/+
	+/w^{1118}; GMR-Gal4, UAS-CHMP2B^{Intron5}/+^{1};UAS-Hook RNAi^{35483}/+
**Figure 5**	*+/w^{1118}; GMR-Gal4, UAS-GFP/+;+/+*
	+/w^{1118}; GMR-Gal4, UAS-GFP/UAS-Ctp^{43115};+/+
	+/w^{1118}; GMR-Gal4, UAS-GFP/UAS-Ctp^{109084};+/+
	+/w^{1118}; GMR-Gal4, UAS-CHMP2B^{Intron5}/+;+/+
	+/w^{1118}; GMR-Gal4, UAS-CHMP2B^{Intron5}/UAS-Ctp^{43115};+/+
	+/w^{1118}; GMR-Gal4, UAS-CHMP2B^{Intron5}/UAS-Ctp^{109084};+/+

Supplementary 4. Genotypes of the flied used in this study.

Reagents	Sources
**Flies**	
GMR-Gal4	Ahmad et al., 2009
UAS-GFP	RRID:BDSC_5431
GMR-Gal4, UAS-GFP	This study
GMR-Gal4, UAS-CHMP2B^{Intron5}	Ahmad et al., 2009
Ik2^{Alice}	RRID:BDSC_31035
UAS-Ik2 RNAi	RRID: BDSC_35266 (Bergalet et al., 2020)
Ird5^{KG08072}	RRID: BDSC_14684
UAS-Spn-F RNAi	RRID:BDSC_65226
Spn-F^{2}	Lin et al, 2015
UAS-GFP-Spn-F	Lin et al, 2015
Hook^{7} and *Hook^{11}*	Kramer et al, 1998
UAS-Hook RNAi lines	VDRC: 35483 AND 35485
UAS-Ctp RNAi lines	VGCR: 43115 and 109084 (Zaytseva et al., 2014)
UAS-CHMP2B^{Intron5}	Ahmad et al., 2009
UAS-ik2	F001016, FlyORF
CS	RRID:BDSC_64349
W^{1118}	RRID:BDSC_5905
**Antibodies**	
anti-synaptotagmin	RRID:AB_2713991
Cy3-Conjugated anti-HRP	RRID:AB_2338959
anti-Rabbit IgG (H+L) Alexa Fluor 488	RRID:AB_2576217

Supplementary 5. Fly lines and reagents used in this study and their sources.

Figure 1	*Chi-squared test for categorical data*	*Summary*	*P value*
	GMR-Gal4,UAS-CHMP2B^{Intron5}/Ik2^{Alice} vs. GMR-Gal4,UAS-CHMP2B^{Intron5}	***	7.58403E-09
	GMR-Gal4,UAS-CHMP2B^{Intron5}/+; UAS-Ik2 RNAi^{235266}/+ vs. GMR-Gal4,UAS-CHMP2B^{Intron5}	***	5.9822E-21
	GMR-Gal4,UAS-CHMP2B^{Intron5}/+; Ird5^{KG08072}/+ vs. GMR-Gal4,UAS-CHMP2B^{Intron5}	ns	0.105646194
Figure 2B	Tukey's multiple comparisons test	Summary	P Value
	Wildtype vs. OK6 > CHMP2B^{Intron5}	**	0.0013
	Wildtype vs. OK6 > mCD8-GFP, CHMP2B^{Intron5}	**	0.0024
	Wildtype vs. OK6 > CHMP2B^{Intron5}, UAS-TBK1	ns	0.5239
	OK6 > CHMP2B^{Intron5} vs. OK6 > mCD8-GFP, CHMP2B^{Intron5}	ns	0.9873
	OK6 > CHMP2B^{Intron5} vs. OK6 > CHMP2B^{Intron5}, UAS-TBK1	ns	0.0543
	OK6 > mCD8-GFP, CHMP2B^{Intron5} vs. OK6 > CHMP2B^{Intron5}, UAS-TBK1	ns	0.0633
Figure 2C	Tukey's multiple comparisons test	Summary	P Value
	Wild type vs. OK6 > CHMP2B^{Intron5}	*	0.0144
	Wild type vs. OK6 > mCD8-GFP, CHMP2B^{Intron5}	*	0.0429
	Wild type vs. OK6 > CHMP2B^{intron5}, UAS-TBK1	ns	0.5674
	OK6 > CHMP2B^{Intron5} vs. OK6 > mCD8-GFP, CHMP2B^{Intron5}	ns	0.9708
	OK6 > CHMP2B^{Intron5} vs. OK6 > CHMP2B^{intron5}, UAS-TBK1	ns	0.1777
	OK6 > mCD8-GFP, CHMP2B^{Intron5} vs. OK6 > CHMP2B^{intron5}, UAS-TBK1	ns	0.3813
Figure 2D	Tukey's multiple comparisons test	Summary	P Value
	Wild type vs. OK6 > CHMP2B^{Intron5}	****	<0.0001
	Wild type vs. OK6 > mCD8-GFP, CHMP2B^{Intron5}	****	<0.0001
	Wild type vs. OK6 > CHMP2B^{intron5}, UAS-TBK1	****	<0.0001
	OK6 > CHMP2B^{Intron5} vs. OK6 > mCD8-GFP, CHMP2B^{Intron5}	ns	0.7216
	OK6 > CHMP2B^{Intron5} vs. OK6 > CHMP2B^{intron5}, UAS-TBK1	ns	0.5821
	OK6 > mCD8-GFP, CHMP2B^{Intron5} vs. OK6 > CHMP2B^{intron5}, UAS-TBK1	ns	0.0665
Figure 2E	Tukey's multiple comparisons test	Summary	P Value
	Wild type vs. OK6 > CHMP2B^{Intron5}	****	<0.0001
	Wild type vs. OK6 > mCD8-GFP, CHMP2B^{Intron5}	****	<0.0001
	Wild type vs. OK6 > CHMP2B^{intron5}, UAS-TBK1	*	0.0129
	OK6 > CHMP2B^{Intron5} vs. OK6 > mCD8-GFP, CHMP2B^{Intron5}	ns	0.9479
	OK6 > CHMP2B^{Intron5} vs. OK6 > CHMP2B^{intron5}, UAS-TBK1	**	0.0051
	OK6 > mCD8-GFP, CHMP2B^{Intron5} vs. OK6 > CHMP2B^{intron5}, UAS-TBK1	***	0.0004
Figure 2F	Tukey's multiple comparisons test	Summary	P Value
	Wild type vs. OK6 > CHMP2B^{Intron5}	****	<0.0001
	Wild type vs. OK6 > mCD8-GFP, CHMP2B^{Intron5}	****	<0.0001
	Wild type vs. OK6 > CHMP2B^{intron5}, UAS-TBK1	**	0.0034
	OK6 > CHMP2B^{Intron5} vs. OK6 > mCD8-GFP, CHMP2B^{Intron5}	ns	0.8572
	OK6 > CHMP2B^{Intron5} vs. OK6 > CHMP2B^{intron5}, UAS-TBK1	ns	0.0785
	OK6 > mCD8-GFP, CHMP2B^{Intron5} vs. OK6 > CHMP2B^{intron5}, UAS-TBK1	**	0.0063
Figure 3	*Chi-squared test for categorical data*	*Summary*	*P value*
	GMR-Gal4,UAS-CHMP2B^{Intron5}/+;Spn-F^{2}/+ vs. GMR-Gal4,UAS-CHMP2B^{Intron5}/+;	***	6.2127E-22
	GMR-Gal4, UAS-CHMP2B^{Intron5}/UAS-Spn-F RNAi^{65226} vs. GMR-Gal4,UAS-CHMP2B^{Intron5}/UAS-GFP	***	1.97234E-39
	GMR-Gal4,UAS-CHMP2B^{Intron5}/Hook^{7} vs. GMR-Gal4,UAS-CHMP2B^{Intron5}	***	0.00001981
	GMR-Gal4,UAS-CHMP2B^{Intron5}/Hook^{11} vs. GMR-Gal4,UAS-CHMP2B^{Intron5}	**	0.005270609
Figure 4	*Chi-squared test for categorical data*	*Summary*	*P value*

	GMR-Gal4,UAS-CHMP2B^{Intron5}/Hook^{11} vs. GMR-Gal4,UAS-CHMP2B^{Intron5}	**	0.005270609
	GMR-Gal4,UAS-CHMP2B^{Intron5}/+;UAS-Hook RNAi^{35483}/+ vs. GMR-Gal4,UAS-CHMP2B^{Intron5}/UAS-GFP	***	9.70605E-10
	GMR-Gal4,UAS-CHMP2B^{Intron5}/+;UAS-Hook RNAi^{35485}/+ vs. GMR-Gal4,UAS-CHMP2B^{Intron5}/UAS-GFP	**	2.02058E-12
Figure 5	*Chi-squared test for categorical data*	*Summary*	*P value*
	GMR-Gal4, UAS-CHMP2B^{Intron5}/UAS-Ctp$^{4311\ 5}$ vs. GMR-Gal4,UAS-CHMP2B^{Intron5}/UAS-GFP	***	0.0000000307
	GMR-Gal4, UAS-CHMP2B^{Intron5}/UAS-Ctp^{109084} vs. GMR-Gal4,UAS-CHMP2B^{Intron5}/UAS-GFP	**	0.0079128247
Supplementary 2	Tukey's multiple comparisons test	Summary	P value
	Wild-type vs. OK6 > +	ns	0.9897
	Wild-type vs. OK6 > GFP, CHMP2B^{Intron5}	**	0.0076
	Wild-type vs. OK6 > CHMP2B^{Intron5}, UAS-IK2	***	0.0001
	OK6 > + vs. OK6 > GFP, CHMP2B^{Intron5}	*	0.0310
	OK6 > + vs. OK6 > CHMP2B^{Intron5}, UAS-IK2	***	0.0010
	OK6 > GFP, CHMP2B^{Intron5} vs. OK6 > CHMP2B^{Intron5}, UAS-IK2	ns	0.7182

Supplementary 6. Values of all the statistical test results.
